# An Exploratory Study of Matrix Metalloproteinase Protein Changes in Unexplained Infertility

**DOI:** 10.3390/life16040676

**Published:** 2026-04-15

**Authors:** Zainab Alhalwachi, Thozhukat Sathyapalan, Stephen L. Atkin, Alexandra E. Butler

**Affiliations:** 1 School of Medicine, Royal College of Surgeons in Ireland, Medical University of Bahrain, Busaiteen 228, Bahrain; satkin@rcsi.com (S.L.A.); or abutler@rcsi.com (A.E.B.); 2 Hull York Medical School, University of Hull, Hull HU6 7RX, UK; thozhukat.sathyapalan@hyms.ac.uk

**Keywords:** matrix metalloproteinase, MMP1, MMP3, MMP17, unexplained Infertility, in vitro fertilization

## Abstract

**Background**: Matrix metalloproteinases (MMPs) have been found to be associated with reproductive complications, including female infertility. This study was conducted to explore the link between MMPs in non-obese women with unexplained infertility (UI) in comparison to women with male factor infertility (MFI) as controls. **Methods**: This pilot exploratory study was carried out on 25 women, 11 with UI and 14 with MFI, undergoing IVF. Blood was drawn on day 21 of the luteal phase. Slow Off-rate Modified Aptamer (SOMA)-scan plasma protein measurement was undertaken for 12 matrix metalloproteinase proteins. Welch’s *t*-test and a permutation test were used to compare group means, and Pearson’s correlations to examine relationships with matrix metalloproteinases. **Results**: No significant differences were seen for baseline demographics and hormonal parameters between the groups, and parameters of the response following IVF did not differ. In the UI group, MMP-3 levels were lower (*p* = 0.045), while MMP-1 and MMP-17 levels were higher (*p* = 0.007 and *p* = 0.010, respectively) compared to the MFI group. In MFI alone, MMP-1 positively correlated with vitamin D_3_ (*p* < 0.05). **Conclusions**: This exploratory study suggests altered circulating MMP-1, MMP-3, and MMP-17 profiles in women with unexplained infertility. These findings require confirmation in larger cohorts and in studies examining endometrial tissue expression and MMP functional activity.

## 1. Introduction

Infertility is the inability to successfully conceive after twelve months of unprotected intercourse and is mostly caused by identifiable abnormalities in males and/or females. Female abnormalities that can lead to infertility include ovulatory dysfunction and anovulation, tubal infertility, endometriosis, or diminished ovarian reserve [1]. In males, infertility can be caused by low testosterone concentrations or low sperm count [2]. Couples may also have multiple factors between them contributing to infertility [3]. When diagnosed, couples can target the abnormality in order to conceive; however, around 30% of infertile couples have unexplained infertility (UI), which is diagnosed when every other abnormality for infertility has been ruled out and is difficult to treat [4]. Studies have attempted to elucidate alternative mechanisms that may contribute to UI.

The matrix metalloproteinase (MMP) family is a group of over 20 zinc-dependent endopeptidases. MMPs have a conserved structure composed of an N-terminal signal peptide, a pro-domain, a catalytic domain, a linker region and a C-terminal hemopexin-like domain. The action of peptide cleavage is due to a glutamate residue within the catalytic motif, and substrate specificity is linked to the C-terminal hemopexin-like domain. MMPs are secreted from cells as zymogens, where the pro-domain and catalytic domain are ligated by a thiol-zn^2+^ interaction, and must undergo further processing for activation [5,6]. MMPs are important for the degradation of various extracellular matrix (ECM) components, such as fibers, proteoglycans, glycoproteins, and polysaccharides, as well as several non-matrix proteins, including, chemokines, and receptors, are classified based on their substrate-specificity and are grouped into: collagenases (MMP-1, MMP-8, MMP-13, MMP-18), gelatinases (MMP-2, MMP-9), stromelysins (MMP-3, MMP-10, MMP-11, MMP-17), matrilysins (MMP-7), membrane-type MMPs (MMP-14, MMP-15, MMP-16, MMP-17), and other MMPs (MMP-12, MMP-19, MMP-20, MMP-21, MMP-22, MMP-23) [7]. The regulation of ECM by MMPs is key for tissue development, maintenance, and remodeling, as well as for cell-to-tissue signaling [8]. In signaling processes, MMPs are important in oocyte release and preparing the endometrium for embryo implantation and successful fertilization [9].

Dysregulation of MMP activity can lead to the development of various diseases related to tissue destruction, fibrosis, and matrix weakening. Elevated MMP-9 expression has been linked to endometriosis [10]. Single-nucleotide polymorphisms in the MMP-7 gene have also been associated with endometriosis risk [11]. Polymorphisms in the MMP-2 gene are linked to protective effects on polycystic ovary syndrome (PCOS) and endometriosis [12]. Elevated MMP-2 has also been linked to endometriosis [13]. Polymorphisms in MMP-1, MMP-2, and MMP-7 are associated with adenomyosis [14,15,16]. MMP-2 and MMP-9 polymorphisms are found to be significantly associated with recurrent spontaneous abortion (RSA) [17]. MMPs also contribute to male fertility, where they play a role in male reproductive processes [18]. Due to their role in remodeling endometrial tissue [19], changes in MMP expression could potentially contribute to mechanisms underlying UI; therefore, we hypothesized that matrix metalloproteinases may have a role in the pathogenesis of UI. The aim of this study was to compare the expression levels of plasma MMPs in patients with UI compared to male factor infertility (MFI) as a control.

## 2. Materials and Methods

### 2.1. Study Design and Participants

Twenty five women were recruited sequentially in this case–control study conducted in 2015 from the IVF Unit, Hull, UK. A total of 11 were recruited as cases of women with unexplained infertility (UI) after undergoing clinical evaluation for excluding all likely causes of infertility, while 14 patients were recruited as presumed fertile, healthy controls from couples with male factor infertility (MFI). Women with UI underwent diagnostic laparoscopy as part of their evaluation. All participants had BMI < 30 kg/m^2^, were in the age range of 20–45 years, and were undergoing IVF treatment. Exclusion criteria included documented immunological or inflammatory disease, infection (acute or chronic), hepatic or renal insufficiency, and diabetes mellitus. Additionally, medical history records showed no prescription or over-the-counter medications were being taken. All patients were non-smokers and had abstained from alcohol for at least six months. Ethical approval was granted by the Yorkshire and The Humber NRES Ethics Committee, UK (approval number 02/03/043) [20].

### 2.2. IVF Protocol

IVF treatment commenced during the subsequent menstrual cycle for all participants using a short antagonist protocol. Recombinant follicle-stimulating hormone (rFSH) stimulation was initiated on day 2 of the cycle with either Merional (Pharmasure, Watford, UK) or Gonal-F (Merck Serono, Feltham, UK). Dosage was tailored individually according to patient AMH levels, antral follicle count, age, and previous ovarian response to treatment. To suppress premature luteinizing hormone (LH) surges, a daily dose of 0.25 mg Cetrotide (GnRH antagonist; Merck Serono, Feltham, UK) was introduced from stimulation on day 6. Final oocyte maturation was triggered when ≥2 leading follicles reached ≥18 mm, using either 0.5 mg Buserelin (Sanofi-Aventis, Frankfurt, Germany) or 5000–10,000 IU human chorionic gonadotrophin (hCG; Pregnyl, Merck Sharp and Dohme, London, UK). Transvaginal oocyte retrieval was carried out 36 h later. To support the luteal phase, vaginal progesterone (Uterogestan, Besins Iscovesco Laboratories, Paris, France; 600 mg nightly) was administered on the day of oocyte retrieval. Embryo transfer was carried out on day 3 or preferably day 5 (blastocyst stage) to maximize implantation potential. Embryo quality was assessed at both cleavage and blastocyst stages using standardized morphological grading criteria [21].

### 2.3. Sample Collection

Before initiation of IVF treatment and before administration of any hormonal therapy, participants underwent mock embryo transfer, as standard clinical practice, and ovarian/endometrial ultrasound at day 21 of the menstrual cycle. Ovulation was detected using serial transvaginal ultrasonography, confirming the presence of the corpus luteum. Fasting blood samples were collected, centrifuged at 3500× *g* for 15 min at 4 °C, and stored at −80 °C for further analysis.

### 2.4. Biochemical and Hormonal Assays

#### 2.4.1. Metabolic Measures

Fasting blood glucose (FBG) levels were measured using a Synchron LX20 analyzer (Beckman-Coulter, HighWycombe, UK). Total cholesterol, triglycerides, and HDL-c were also quantified enzymatically using a Synchron LX20 analyzer (Beckman-Coulter). Low-density lipoprotein cholesterol (LDL-c) was calculated using the Friedewald equation [22]. Serum insulin concentrations were determined by competitive chemiluminescent immunoassay (Immulite 2000, Euro/DPC, Llanberis, UK). Insulin resistance was assessed using the HOMA-IR, calculated as (insulin × glucose)/22.5 [23]. Glycated hemoglobin (HbA1c) was measured by ion-exchange chromatography. CRP was evaluated using enzymatic assays on the Synchron LX20 analyzer (Beckman-Coulter). WBC was measured with a Beckman Coulter counter (Beckman Coulter).

#### 2.4.2. Reproductive Hormones

To determine AMH levels, an immunoenzymatic assay (Beckman-Coulter) was used. Circulating androgens were quantified by liquid chromatography–tandem mass spectrometry (LC/MS/MS; Acquity UPLC-Quattro Premier XE-MS, Waters, Manchester, UK). Sex hormone-binding globulin (SHBG) was assessed using an immunometric fluorescence assay (Immulite 2000 analyzer; upper limit 2.0 nmol/L). The Free Androgen Index (FAI) was calculated according to the formula (testosterone/SHBG) × 100. Thyroid hormone levels, TSH, Free-T3, and Free-Thyroxine (Free-T4) were determined using an immunoassay on the Abbott Architect i4000 platform (Abbott Diagnostics, Maidenhead, UK).

#### 2.4.3. Matrix Metalloproteinase Quantification

Matrix metalloproteinase proteins were quantified using the Slow Off-rate Modified Aptamer (SOMAscan) platform, version 3.1 (SomaLogic, Boulder, CO, USA), as previously described [24,25]. In brief, synthetic SOMAmers with fluorescent labeling were first bound to analyte/primer beads. The resulting complexes were immobilized on a streptavidin matrix. Ultraviolet (UV) light was applied to cleave the photocleavable linker, causing the release of the analyte–SOMAmer complexes into the solution. These complexes were subsequently re-immobilized on a streptavidin matrix through analyte-mediated biotinylation. After which, the SOMAmers were eluted and used as surrogates for protein quantification. Quantification was achieved through hybridization with complementary oligonucleotides, ensuring accurate signal detection. Calibration standards were included, and normalization procedures (comprising hybridization control, median signal scaling, and calibration signal correction) were applied in accordance with established protocols: intra-plate CV: plasma ~3.6%; inter-plate CV: plasma ~3.8%; and total CV: plasma ~5.3% [26,27]. Proteins analyzed were matrix metalloproteinase-1 (MMP-1), matrix metalloproteinase-2 (MMP-2), matrix metalloproteinase-3 (MMP-3), matrix metalloproteinase-7 (MMP-7), matrix metalloproteinase-8 (MMP-8), matrix metalloproteinase-9 (MMP-9), matrix metalloproteinase-10 (MMP-10), matrix metalloproteinase-12 (MMP-12), matrix metalloproteinase-13 (MMP-13), matrix metalloproteinase-14 (MMP-14), matrix metalloproteinase-16 (MMP-16), and matrix metalloproteinase-17 (MMP-17).

### 2.5. Statistical Analysis

As this was an exploratory pilot, no formal power calculation was performed; however, *n* = 25 was selected to estimate effect sizes for future studies. The mean ± standard deviation (SD) was presented as descriptive data for continuous data. For the values of matrix metalloproteinases and metabolic or hormonal levels, normality was assessed using the Kolmogorov–Smirnov (K-S) statistical test; for all measurements, the K-S *p*-value was >0.05, indicating that the data were normally distributed. Welch’s *t*-test was used to compare the differences between groups, and the result was determined to be statistically significant if *p* < 0.05. To account for the small sample size, a two-fold permutation test was conducted with 10,000 permutations (*p* < 0.05). Associations between protein levels and metabolic and hormonal parameters were calculated by Pearson’s correlations. For statistical analysis, Graphpad Prism v9.5.1 (San Diego, CA, USA) was utilized.

## 3. Results

### 3.1. Patient Characteristics

There were no significant differences found between the MFI and UI groups regarding age, body mass index (BMI), Homeostatic Model Assessment of Insulin Resistance (HOMA-IR), and serum lipids. C-reactive protein (CRP) and white blood cell count (WBC), as indicators of inflammation and infection, were within the normal range and similar for both MFI and UI. There were no significant differences found between the MFI and UI groups with regard to levels of reproductive hormones, number of positive pregnancy tests, eggs retrieved, and embryos created, including the G3D3, which are the top-quality embryos at day 3 as per the Alpha consensus [21]. Additionally, fertility rates and live birth rates did not significantly differ between the two groups (Table 1).

### 3.2. Matrix Metalloproteinases

**Figure 1:**
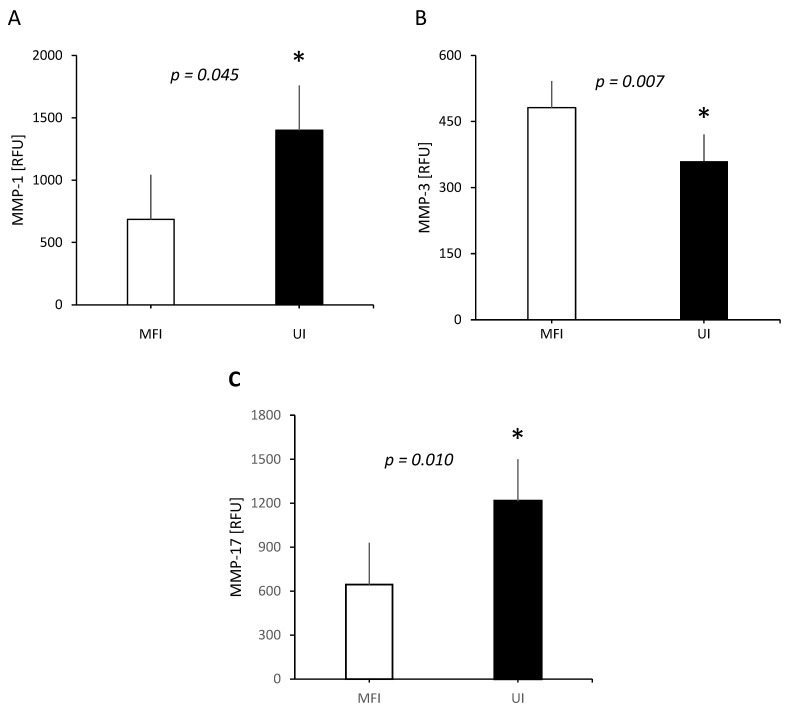
Matrix metalloproteinases that differed between male factor infertility (MFI) and unexplained infertility (UI). MMP-1 (*p* = 0.045) (**A**) was higher in the UI cohort, MMP-3 (*p* = 0.007) (**B**) was lower in the UI cohort, and MMP-17 (*p* = 0.010) (**C**) was higher in the UI cohort. * *p* < 0.05. Data is presented as mean (SD). Protein levels are reported as Relative Fluorescence Units (RFU).

Significantly decreased expression level and high effect sizes was seen for MMP-3 in the UI group compared to MFI (359.8 RFU vs. 481.2 RFU, *p* = 0.007, Cohen’s d = −1.16), whilst conversely, MMP-1 was significantly elevated in UI compared to MFI (1401.2 RFU vs. 685.4 RFU, *p* = 0.045, Cohen’s d = 0.94). No statistical significance was found for MMP-2, MMP-7, MMP-8, MMP-9, MMP-10, MMP-12, MMP-13, MMP-14, MMP-16, or MMP-17. When confirming results with a permutation test, the analysis with 10,000 permutations revealed a statistically significant difference between the UI and MFI for MMP1 (*p* = 0.010), MMP3 (*p* = 0.007), and MMP17 (*p* = 0.010), as shown in Table 2 and Figure 1.

### 3.3. Correlation Analysis

**Figure 2:**
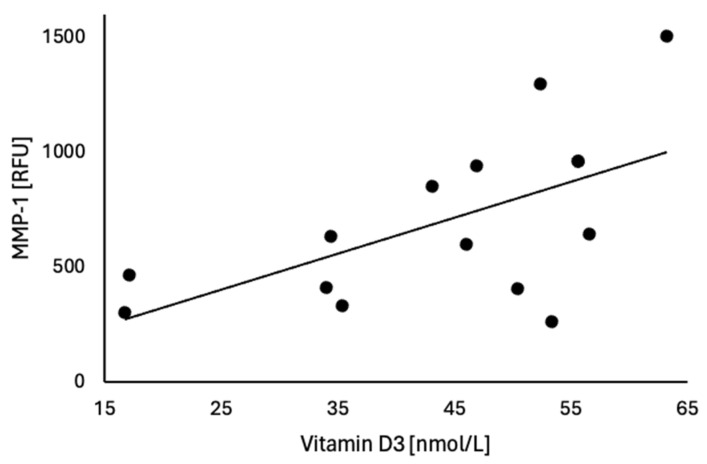
Significant correlations between matrix metalloproteinase-1 (MMP-1) and vitamin D_3_ in male factor infertility (MFI) (r = 0.58, *p* = 0.028). Protein level is reported as Relative Fluorescence Units (RFU) and vitamin D_3_ level is reported as nanomoles per liter (nmol/L).

We examined relationships between statistically significant matrix metalloproteinases and biochemical and hormonal parameters. In the MFI cohort, there was a significant positive correlation between MMP-1 and vitamin D_3_ (*r* = 0.58, *p* = 0.028) as shown in Figure 2.

## 4. Discussion

Matrix metalloproteinases are responsible for protein degradation for extracellular matrix components, as well as non-extracellular proteins, and play an important role in maintaining differing physiological functions, such as embryogenesis, morphogenesis, angiogenesis and wound repair [28]. In fertility, remodeling of the endometrium extracellular matrix is important for embryo implantation, trophoblast invasion, and placentation to ensure successful fertilization [9]. Dysregulation of this reproductive process could have a role in causing infertility. Reproductive issues tend to have diagnosable, known causes that can be targeted for treatment; though for women with unexplained infertility, that cause is not determined. Exploring the association of MMPs in reproduction could potentially provide a clinical target for infertility correction. In this comprehensive study, MMP expression levels were explored to elucidate their role in unexplained infertility. Of the 12 MMPs studied, MMP-1 and MMP-17 were found to be significantly higher in UI patients when compared to the MFI control group. Furthermore, MMP-1 was positively correlated with vitamin D_3_ in the MFI group only. MMP-3 was significantly lower in the UI group versus the MFI group. No correlation was found between MMP-1 or MMP-3 in the UI group with vitamin D_3_, follicle-stimulation hormone, or luteinizing hormone.

MMP-1 is a collagenase that, upon stimulation, is secreted by multiple cells, including epithelial cells, fibroblasts, and macrophages [29]. Under normal conditions, MMP-1 expression in the endometrium changes with the progression of the menstrual cycle and is important for embryo implantation [30]. Unregulated changes in MMP-1 could affect the endometrium and lead to reproductive disorders that may affect fertility; however, results have been inconsistent. A study carried out in patients with polycystic ovary syndrome (PCOS) showed no difference in MMP-1 levels in the follicular fluid; however, there was a significant decrease in tissue inhibitor of metalloproteinase-1 (TIMP-1), an MMP-1 inhibitor [31]. Case–control studies have found that the *-1607 1G/2G* single-nucleotide polymorphism (SNP) in the MMP-1 gene, which enhances gene transcription, is associated with an increased risk of PCOS, but no association was found with idiopathic recurrent spontaneous abortion (IRSA) [32,33]. Wang et al. examined the expression of MMP-1 in poor prognosis embryos but did not find significant changes in expression levels, although there was an increased expression of TIMP-1 in later preimplantation developmental stages [34]. In endometriosis, Ye et al. found a significant association between the MMP-1 *-1607 1G/2G* SNP and endometriosis [35]. However, Eugeniu et al. did not find a significant change in MMP-1 expression in patients with endometriosis [36]. Although the data are inconsistent, it is important to note that MMP-1 function may differ due to various regulatory mechanisms, including TIMPs, pro-inflammatory cytokines, growth factors, and ECM components. Pino et al. found an increase in MMP-1 levels in endometrial cells upon stimulation with tumor necrosis factor (TNF) [37]. Furthermore, in the endometrium, macrophages were found to be abundant in patients with endometriosis [38], suggesting a role for the inflammatory response as a mechanism of activation for MMP-1. Studies have also shown an association between patients with UI and increased cytokine expression such as interleukin-2 (IL-2), tumor necrosis factor-a (TNF-a), and interleukin-8 (IL-8), amongst others [39,40,41]. Our data aligns with this theory, although further investigation into the expression of cytokine markers in these patients in relation to MMP-1 expression levels and function is needed. In this study, MMP-1 was positively correlated with vitamin D_3_ in the MFI cohort alone. Some studies have shown an association between vitamin D_3_ and fertility [42]. Furthermore, it has been found that vitamin D significantly downregulates MMP-2 and MMP-9, suggesting that vitamin D has a protective role in fertility through various mechanisms, including MMPs. The apparent lack of protective effect of vitamin D in our study suggests a disruption in the pathway and further studies are needed to fully understand the mechanism.

MMP-17 is a membrane-type 4 matrix metalloproteinase, also known as MT4-MMP, anchored to the cellular membrane by glycosylphosphatidylinositol anchor (GPI) and is expressed prominently in the organs such as the uterus and brain and transiently in reproductive organs and stomach [43]. MT4-MMP is also found in monocyte/macrophage cell lines and displays pro-inflammatory activity from its role in the cleavage of pro-TNFa [44]. While there are no studies directly linking the role of MT4-MMP to infertility, the increased expression levels of MT4-MMP and MMP-1 suggest a potential pro-inflammatory pathway that needs to be determined and further explored in future studies.

Like MMP-1, MMP-3 plays a vital role in the regulation of the endometrium ECM to support fertilization and embryonic development [45]. MMP-3, also known as stromelysin-1, is produced by various cells including endothelial cells and digests a range of ECM proteins, including pro-MMPs [30]. It has been shown that the activation of the MMP-3 signaling pathway results in the transcription of both MMP-3 and MMP-1 genes [46]. Furthermore, MMP-3 acts as a zymogen activator and converts pro-MMP1 to MMP-1 [47]. While MMP-1 and MMP-3 have similar activation pathways, they do not necessarily upregulate equally. For example, Genevay et al. found in disc herniation that expression levels of MMP-1 increased while that of MMP-3 decreased [48]. However, in a study on corneal epithelial cells, both MMP-1 and MMP-3 were upregulated by cytokine stimulation [49]. Similarly, our data showed an inverse relation between MMP-1 and MMP-3, where MMP-1 was significantly increased while MMP-3 was significantly decreased in unexplained infertility. Studies have found an association between rs35068180 MMP-3 SNP and recurrent pregnancy loss (RPL) [50,51]; furthermore, this genetic variation results in increased expression of MMP-3 [52]. Palmieri et al. found increased MMP-3 expression in women with endometriosis [53]. Similarly, a study in women with unexplained infertility found that MMP-3 gene expression was decreased in the women who became pregnant after assisted reproductive treatment compared to the women who did not become pregnant [54]. This data suggests that increased MMP-3 increases the risk of infertility. Our data conflicts with the results described here, where we witnessed a decrease in MMP-3 expression levels in women with unexplained infertility. The difference shown could potentially be explained by the differential expression of MMP-3 that is regulated by a large network of cellular and extracellular components, one of which is progesterone. Progesterone is known to play an important role in preparing the endometrium for embryo receptiveness [55,56], particularly in prompting decidualization, where stromal cells differentiate into large secretory epithelioid cells [57]. Subsequently, these epithelioid cells secrete MMPs to regulate the ECM for embryo implantation and successful fertilization [58]. Decreases in progesterone caused by progesterone resistance has been linked to decreased MMP expressions, including MMP-3 [58]. Studies have also associated reduced progesterone to reproductive complications, including women with UI [59,60]. Further studies are needed to determine a mechanistic association between reduced progesterone production or increased progesterone resistance and decreased MMP-3 expression levels that cause unexplained infertility. Another potential mechanism that needs to be elucidated by future studies is decidual macrophage dysfunction. Decidual macrophages were found to secrete MMPs, with MMP-3 being the most predominant, and are linked to ECM maintenance for successful pregnancy [61]. Additionally, it has been shown in mice that dysfunctional decidual macrophages negatively impact successful pregnancy rates [62]. It is possible that decidual macrophages are dysfunctional in women with unexplained infertility, which potentially leads to decreased MMP-3 and dysregulated ECM for fertilization. A limitation of this study is the reliance on circulating levels as opposed to tissue levels of MMPs. However, circulating MMP levels are used as substitute biomarkers for preliminary assessments of infertility and further studies on tissue-specific MMP levels are needed to draw effective conclusions.

Infertility may be caused by a combination of both male and female reproductive issues [63]. In this study, clinical evaluation was undertaken to exclude identifiable female causes of infertility, so that only a significant male factor was present in the MFI group. These women were considered clinically normal from a female reproductive perspective. This group was then used as the control subjects, and investigation of the UI group, including diagnostic laparoscopy, gave no indication as to why they could not conceive. Although women in the MFI group had partners with significant male infertility, a subtle female factor cannot be completely excluded. The strengths of this study lie in the well-matched MFI and UI group sample for age and BMI. In addition, all these women were undergoing an IVF program for fertility; none had been on any hormonal contraception, they had all stopped any alcohol consumption, and all were non-smokers. So, these confounders were excluded from the analysis. It is well recognized that endothelial function varies throughout the menstrual cycle, and this confounder was mitigated by including only women at day 21 of their cycle [64]. Limitations include the study’s small sample size, and a type 2 statistical error may indicate statistical significance with a larger cohort. Based on MMP1, the marker showing the largest observed standardized difference between UI and MFI (Cohen’s d = 0.99, 95% CI 0.14–1.82). Under a two-sided independent-samples *t*-test with α = 0.05 and 80% power, this corresponded to 18 participants per group; however, sensitivity analyses allowing for 20–30% variance inflation suggested that 25–30 participants per group would represent a more conservative and robust target for future validation. Only a Caucasian population was used, and the study needs to be repeated with a more ethnically diverse population. The only difference in the investigation between the MFI and the UI subjects was that the UI subjects had a diagnostic laparoscopy whilst the MFI subjects did not. In addition, we measured the plasma MMPs that may not reflect what is happening at the tissue level.

In conclusion, this exploratory study suggests altered circulating MMP-1, MMP-3, and MMP-17 profiles in women with unexplained infertility. These findings require confirmation in larger cohorts and in studies examining endometrial tissue expression and MMP functional activity.

## Figures and Tables

**Table 1:** Demographic and biochemical data compared between male factor infertility (*n* = 14) and unexplained infertility (*n* = 11). The data is presented as mean (SD). The *p* values were calculated using Welch’s *t*-test to determine any differences between the groups.

	MFI (*n* = 14)	UI (*n* = 11)	*p* Value
Age (years)	32.6 (4.0)	33.8 (5.3)	0.51
Minimum	24	25	
Maximum	38	42	
BMI (kg/m^2^)	25.7 (2.6)	25.3 (4.9)	0.84
Minimum	19.2	19	
Maximum	28.2	33.1	
HOMA-IR	1.5 (0.7)	1.9 (1.4)	0.45
Cholesterol (mmol/L)	4.8 (0.8)	4.6 (0.7)	0.44
Triglycerides (mmol/L)	0.9 (0.5)	1.0 (0.4)	0.67
HDL-c (mmol/L)	1.6 (0.4)	1.6 (0.2)	0.97
LDL-c (mmol/L)	2.8 (0.7)	1.0 (0.4)	0.24
CRP (mg/L)	1.9 (1.3)	2.4 (2.0)	0.51
WBC 10^9^/L	5.9 (1.7)	7.2 (2.2)	0.12
AMH (ng/mL)	22.4 (15.3)	24.5 (12.5)	0.72
FAI	1.4 (0.7)	0.8 (0.9)	0.11
TSH (mU/L)	2.3 (1.2)	2.0 (0.8)	0.53
Free-T3 (pmol/L)	4.7 (0.7)	4.8 (0.6)	0.66
Free-T4 (pmol/L)	11.2 (1.6)	11.4 (0.9)	0.66
Positive pregnancy test	0.3 (0.5)	0.3 (0.5)	0.95
Number of eggs retrieved	9.0 (7.5)	8.4 (3.2)	0.78
Number of embryos created	3.7 (3.0)	5.2 (2.4)	0.18
G3D3	3.4 (2.2)	2.7 (2.6)	0.49
Fertility rate	0.6 (0.2)	0.6 (0.4)	0.89
Top quality embryo (proportion)	0.3 (0.2)	0.4 (0.4)	0.36
Live birth rate	0.0 (0.0)	0.0 (0.0)	1.00

BMI, body mass index; HOMA-IR, Homeostatic Model Assessment of Insulin Resistance; HDL-c, High-Density Lipoprotein Cholesterol; LDL-c, Low-Density Lipoprotein Cholesterol; CRP, C-Reactive Protein; WBC, white blood cell count; AMH, Anti-Müllerian Hormone; FAI, Free Androgen Index; TSH, thyroid-stimulating hormone; Free-T3, Free-triiodothyronine; Free-T4, Free-Thyroxine; and G3D3, top-quality day 3 embryos.

**Table 2:** Comparison of plasma levels of matrix metalloproteinases in male factor infertility (MFI) and unexplained infertility (UI) (Mean ± Standard Deviation (SD)). Protein levels are reported as Relative Fluorescence Units (RFUs).

	UI Mean ± SD	MFI Mean ± SD	Cohen’s d	95% CI	*p* Value	Permutation Test *p* Value
MMP-1	1401.2 ± 1010.9	685.4 ± 380.4	0.99 *	[0.15, 1.82]	0.045 *	0.010 *
MMP-2	6477.6 ± 1717.7	7109.9 ± 702.6	−0.51	[−1.31, 0.30]	0.272	0.232
MMP-3	359.8 ± 74.6	481.2 ± 127.6	−1.13 *	[−1.98, −0.28]	0.007 *	0.007 *
MMP-7	968.8 ± 318.7	1103.8 ± 289.0	−0.45	[−1.25, 0.35]	0.286	0.269
MMP-8	3806.9 ± 3822.6	2575.1 ± 886.9	0.47	[−0.33, 1.27]	0.318	0.266
MMP-9	39,479.1 ± 22,596.5	25,692.4 ± 14,395.1	0.75 *	[−0.07, 1.57]	0.097	0.071
MMP-10	631.1 ± 310.7	820.9 ± 248.1	−0.69	[−1.50, 0.13]	0.115	0.102
MMP-12	848.9 ± 558.4	969.1 ± 257.6	−0.29	[−1.08, 0.50]	0.520	0.482
MMP-13	584.9 ± 519.9	636.3 ± 632.7	−0.09	[−0.88, 0.70]	0.826	0.773
MMP-14	923.6 ± 449.4	1957.5 ± 2484.6	−0.55	[−1.35, 0.26]	0.149	0.267
MMP-16	717.0 ± 295.1	601.8 ± 226.8	0.45	[−0.35, 1.24]	0.298	0.346
MMP-17	1215.5 ± 971.1	645.1 ± 74.0	0.89 *	[0.06, 1.71]	0.080	0.010 *

* *p* < 0.05. MMP-3, 7–9, 10, 12–14, 16, 17, matrix metalloproteinase 1–3, 7–9, 10, 12–14, 16, 17.

## Data Availability

The data that support the findings of this study are available from the corresponding author upon reasonable request.
